# Association of prophylactic mannitol administration with perihemorrhagic edema and long-term outcome in patients with intracerebral hemorrhage

**DOI:** 10.1186/s12883-026-04858-w

**Published:** 2026-04-01

**Authors:** Maximilian Kaertner, Kosmas Macha, Jochen Sembill, Anne Mrochen, Michael Knott, Stefan Lang, Hannes Lücking, Joji B. Kuramatsu, Bastian Volbers

**Affiliations:** 1 Department of Neurology, Friedrich-Alexander-Universität Erlangen-Nürnberg (FAU), Erlangen, Germany; 2 Department of Neuroradiology, Friedrich-Alexander-Universität Erlangen-Nürnberg (FAU), Erlangen, Germany; 3 Department of Neurology, Inselspital, Bern University Hospital, University of Bern, Bern, Switzerland; 4 Department of Neurology, RoMed Klinikum Rosenheim, Rosenheim, Germany

**Keywords:** Intracerebral hemorrhage, perihemorrhagic edema, osmotherapy, mannitol, outcome

## Abstract

**Background:**

Perihemorrhagic edema (PHE) is an independent predictor of functional outcome after intracerebral hemorrhage (ICH). Although smaller studies have shown an association between prolonged prophylactic mannitol administration (PPMA) and PHE volume reduction, its therapeutic effect remains unclear.

**Methods:**

We included patients with spontaneous ICH from the UKER-ICH database. The primary outcome was defined as the change in PHE volume from admission to its maximum extent (PHE growth). We used linear regression models adjusted for peak ICH volume to estimate associations of PPMA with PHE, and multivariable logistic regression models to evaluate functional outcome (day-90; modified Rankin-Scale 4–6 = poor).

**Results:**

826 patients were included. Ninety-five patients received mannitol. The treated patients had larger median peak ICH volumes (42 (IQR 19.2–70.4) vs. 11.7 (IQR 4.6–27.7) mL, *p* < 0.001). Linear regression demonstrated an association between PPMA and increased PHE growth and peak PHE volume (adjusted b = 6.2, 95%CI 2.16–10.63 and b = 8.4, 95%CI 3.16–13.6, respectively). After PS-matching (*n* = 116), the peak ICH volume was evenly distributed, while PPMA was associated with larger median peak PHE volumes (45.7 (IQR 24-78.2) vs. 35.2 (IQR 19.7–52.7) mL, *p* < 0.05) and growth (21.5 (IQR 9.2-42-8) vs. 11.9 (IQR 1.4–25.8) mL, *p* < 0.05). PPMA was associated with poor outcome (adjusted OR = 2.09, 95%CI 1.01-4.00; PS-matched cohort (*n* = 114): 45 (79%) vs. 36 (65%), *p* = 0.097 (trend)).

**Conclusions:**

We found no association between PPMA and reduced PHE volume or improved outcomes, and cannot exclude potential harm. However, we also cannot exclude a potential benefit of short-term mannitol administration up to 72 h after onset in reducing PHE volume.

**Clinical trial number:**

https://www.clinicaltrials.gov/study/NCT03183167 (2017-06-09)

## Introduction

Perihemorrhagic edema (PHE) is an imaging surrogate marker for detrimental secondary inflammatory processes after ICH and an established independent predictor of patient outcome [1–5]. In particular, rapid PHE expansion within the first 72 h after ICH ictus [2–4, 6, 7] and peak PHE volume [4] are considered edema measures associated with poor outcome. However, effective therapeutic approaches addressing PHE development are still lacking. Thus, an increased focus has been placed on treatment options targeting PHE growth [8]. Based on its mechanism of action - establishing an osmotic gradient that promotes the movement of water from the cerebral extracellular space into the vasculature [9], the administration of mannitol in repeated boluses is recommended to attenuate cerebral edema formation in case of a severe ischemic stroke, with the aim of preventing further increases in intracranial volume [10]. However, evidence regarding the efficacy of this approach in patients with intracerebral hemorrhage remains inconclusive and is therefore a matter of ongoing debate [10, 11]. In particular, it has been discussed whether the blood–brain barrier disruption described after spontaneous ICH [1] may lead to a progressive parenchymal accumulation of mannitol, potentially exacerbating the development of perihematomal edema in affected patients [9, 12]. Thus, although current European and American guidelines suggest mannitol as a possible bridge-to-therapy treatment for acute intracranial pressure (ICP) elevations, no precise recommendations are given regarding its prophylactic use in treating PHE growth [13–15]. Furthermore, evidence of treatment efficacy regarding functional outcome is poor and still under debate [10, 11]. In this study, we aimed to investigate the associations of prolonged prophylactic mannitol administration over several days with long-term outcome and PHE development.

## Methods

### Patient selection

We retrospectively investigated patient data from our prospectively organized institutional database (*N* = 1322; UKER-ICH, NCT03183167). ICH was defined as spontaneous if it was related to cerebral amyloid angiopathy or hypertension. Exclusion criteria included surgical hematoma evacuation or decompressive hemicraniectomy, therapeutic hypothermia, ICH due to vascular malformation, sinus thrombosis or systemic thrombolysis, ICH associated with trauma, tumors, and epidural or subdural hemorrhage. Additionally, patients with insufficient data on cerebral imaging and treatment protocols or early withdrawal of care (< 24 h) were excluded.

### Neuroimaging and volumetric assessment of ICH and PHE volume

A fourth-generation CT scanner (Somatom 64 or Somatom AS+, Siemens Healthcare, Erlangen) was used for the CT scans. Each scan consisted of either a multislice spiral CT dataset, or 10–12 slices of 4.8 mm thickness for the skull base and 10–12 slices of 7.2 mm thickness for the cerebrum (Somatom 64), or 22–25 slices of 4.8 mm thickness for the entire brain (Somatom AS+) in the axial plane. We applied a validated threshold-based semiautomatic volumetric algorithm [16] to assess the ICH and PHE volumes. In brief, a manual region of interest (ROI) was drawn. PHE was identified automatically on the basis of a threshold range of 5–33 Hounsfield units (HU) within the ROI. For hematoma identification, a threshold range of 44–100 HU was used. ICH and PHE volumes [mL], GRAEB scores [17] and intraventricular hemorrhage (IVH) at baseline and at any follow-up CT scan including the time of the respective neuroimaging were recorded. The peak volume or value of a variable was defined as the maximum volume or value measured during the hospital stay. Early secondary hematoma expansion was defined as any increase in the ICH volume between admission and follow-up imaging within the first 72 h after admission. Due to the retrospective design, CT scans had to be merged into time clusters (days 1, 2–3, 4–6, 7–9, and 10–12) for better comparison of PHE development [4]. For the analysis, the maximum volume or measurement value of each time cluster was used.

### Procedures and patient treatment

Patients were treated in accordance with existing guidelines [14, 15]. In the acute setting, timely achievement of the blood pressure target < 140/90mmHg and timely reversal of any anticoagulation treatment were the aims. Follow-up imaging was performed in case of secondary deterioration or in order to monitor ICH and PHE volumes due to the treating physician. Intensive care unit treatment was preferred if any surgical intervention was planned or if mechanical ventilation was necessary. An extraventricular drain (EVD) was applied in joint decision with the attending neurosurgeon in the case of an obstructive hydrocephalus or a relevant intraventricular blood volume. The decision to perform decompressive surgery or hematoma evacuation was due to the treating physician. The decision regarding prophylactic mannitol administration was made by the treating physician as early as possible (usually after a follow-up imaging on the second day after admission) considering baseline and follow up PHE and ICH volume as well as patient age and baseline mRS. A total of 250 mL of 20% mannitol was applied 4 times/day. We defined “prophylactic” osmotic therapy as osmotic treatment to prevent life-threatening PHE and increased intracranial pressure before an intracranial pressure crisis or herniation was imminent or present. The target serum osmolality level was 320 mosm/kg. In general, mannitol was tapered after an administration period of 6 days over a period of 5–7 days. In selected patients, mannitol was continued in case of ongoing elevated ICP (> 20mmHg) or if a high risk of herniation was suspected. Serum osmolality was measured directly after each administration of mannitol. The mannitol dosage was reduced to 125 mL of 20% mannitol if the serum osmolality was > 320 mosm/kg, and paused if the serum osmolality was > 325 mosm/kg. If the target range could not be achieved, additional doses were applied up to a maximum of 6 doses per day. An alternative osmotic treatment involving continuous intravenous administration of hypertonic saline (3%, HS) with a target serum sodium concentration of 150mmol/L was considered due to the treating physician in addition to the mannitol treatment or as a single osmotic treatment [18].

### Outcomes

The primary outcome was absolute PHE growth up to peak volume (defined as the difference between peak PHE volume and admission PHE volume). Secondary outcomes included absolute peak PHE volume, PHE growth up to peak volume per day (defined as PHE growth up to peak PHE volume divided by the number of days between peak PHE volume and admission PHE volume) [7], as well as PHE growth between the predefined time clusters days 2–3, days 4–6, days 7–9 and days 10–12 to analyze time-dependent associations with edema evolution following the initiation of mannitol therapy [9]. In addition, the functional outcome on day 90 dichotomized into favorable (mRS score 0–3) and poor (mRS score 4–6) outcome was included as secondary outcome [19]. Functional outcome was assessed on the basis of the modified Rankin Scale score (mRS) by a trained and certified rater.

### Statistical analysis

We performed the statistical analysis using the IBM SPSS Statistics 29 software package (spss.com; IBM, Armonk, New York, USA). The two-sided α-level was set at 0.05. The ICH and PHE volume data were not normally distributed according to the Kolmogorov-Smirnov and Shapiro-Wilk-Tests. Data are presented as numbers and percentages (%, statistical test: Chi-Square test), and median and interquartile range (statistical test: Mann-Whitney-U-Test). We assessed the effect of mannitol on PHE growth up to peak volume and peak PHE volume in the overall cohort using multivariable linear regression models adjusted for peak ICH volume to account for imbalances in hematoma volume as a relevant confounder for peak PHE volume and PHE growth [4]. In a sensitivity analysis, we excluded patients receiving HS to avoid possible confounding caused by additional osmotic treatment. In addition, we included 1)mannitol treatment, 2)serum osmolality and 3)the serum osmolar gap as an independent variable and 1)peak PHE volume, 2)the maximum PHE volume of each time-cluster as well as 3)the difference in PHE volume between two consecutive time-clusters as the dependent variable in a linear regression model adjusted for peak ICH volume to assess a possible dose- and time-dependent [9] association of mannitol administration with edema development. Patients treated with HS were excluded from this analysis. Since mannitol dosing was adapted on the basis of serum osmolality, and the serum osmolar gap correlates to some extent with mannitol serum concentrations [20], we used those two measures as proxies of mannitol associated osmotic activity.

The effect of mannitol on long-term outcome was estimated using a multivariable binary logistic regression model adjusted for age, baseline mRS, peak ICH volume, peak PHE volume, use of hypertonic saline and peak GRAEB-score in the overall cohort and in a separate model adjusted for deep and lobar locations [21]. We performed a sensitivity analysis to assess the association of prophylactic mannitol use with poor outcome in patients with large and small peak hematoma volumes using a cutoff of 20 mL as well as a sensitivity analysis excluding patients with a baseline mRS score > 3. The baseline mRS score was defined as the mRS score during the week before symptom onset.

In addition, owing to significant differences in baseline characteristics between the mannitol and non-mannitol groups, a propensity score (PS) matching was performed based on a logistic regression as an estimation algorithm (exact matching, 1:1 ratio, caliper 0.1). For the assessment of PHE development (primary outcome), peak ICH volume and age were used as matching parameters (PS Match Edema), which are both considered to be associated with peak PHE volume [5, 22]. To compare long-term clinical outcomes (secondary outcome) between treatment groups, we performed a PS match accounting for the established outcome predictors of peak ICH volume, age, baseline mRS and baseline NIHSS [23, 24] (PS Match Outcome). For both PS matches (Edema and Outcome), patients who received HS and who suffered brainstem hemorrhage were excluded in order to avoid confounding by an additional osmotic therapy and location related associations with outcome [21]. Additionally, the number of CT scans and the time point of the last CT-scan were used as additional matching parameters for both PS Match Edema and PS Match Outcome to account for imbalances regarding the period of available neuroimaging, which is considered to be a relevant confounder for peak PHE volume and PHE growth as well as its association with outcome [4]. Missing data led to exclusion in the respective analysis.

## Results

The UKER-ICH database includes 1322 patients in total (all ICH patients admitted between 01/2006 and 12/2015). Among those, 496 patients were excluded due to intraventricular hemorrhage only (*N* = 21), surgical hematoma evacuation or decompressive hemicraniectomy (*N* = 74), therapeutic hypothermia (*N* = 60), sinus thrombosis (*N* = 9) or systemic thrombolysis (*N* = 1), subarachnoid hemorrhage (*N* = 2), and traumatic ICH and/or epidural or subdural hemorrhage (*N* = 7). No patients were excluded due to a vascular malformation. Furthermore, patients with insufficient imaging data (*N* = 108), insufficient data regarding therapeutic regimens (*N* = 22) and early (< 24 h) withdrawal of care (*N* = 192) were excluded. Data on long-term outcome were missing for 54 patients in the overall cohort and for two patients in the PS-matched subgroup. Therefore, these patients were excluded from the analysis of long-term outcomes.

### Patient and imaging characteristics (overall cohort)

Tables 1 and 2 show detailed patient and imaging characteristics in the overall cohort and those differentiated by mannitol treatment. In total, 826 patients (459 male (44%)) were included. A total of 341 patients (41%) presented with lobar ICH, 386 (47%) with deep ICH and 99 (12%) with infratentorial ICH (5 patients with deep location and additional brainstem extension were defined as deep for the analyses). Ninety-five patients were treated with mannitol. Median treatment initiation was on day 2 after admission (IQR 1–3) with a median duration of 7 days (IQR 5–10) without tapering. There were several imbalances between patients with and without mannitol treatment relevant for PHE evolution as well as outcome prediction (see Tables 1 and 2): Patients treated with mannitol were significantly younger and more often presented with additional intraventricular hemorrhage. Furthermore, they showed larger baseline and peak ICH and PHE volumes, a greater early secondary hematoma expansion volume and a higher baseline NIHSS score. The peak ICH and PHE volumes occurred later in the mannitol group. The median mRS score on day 90 and the in-hospital mortality rate were higher in patients treated with mannitol than in patients without mannitol treatment. Thirty-four patients received HS in addition to mannitol and 14 patients received HS as osmotic treatment in the non-mannitol group.

**Table 1:** Clinical and patient characteristics of the overall cohort and those differentiated by mannitol treatment. Long-term outcome data were available for 772 patients. Data are given as n (%), median (interquartile range). NIHSS: National Institute of Health Stroke Scale. mRS: modified Rankin Scale. GCS: Glasgow Coma Scale [25]. *Refers to the day after admission (admission = day 1). ^†^Refers to days of treatment. P-values refer to comparisons between patients with and without mannitol treatment

	Whole Cohort (*n* = 826)	No Mannitol (*n* = 731)	Mannitol (*n* = 95)	*P* Value
Demographics				
Age [years]	73 (63–80)	74 (64–80)	70 (59–77)	0.004
Male [n (%)]	459 (56%)	404 (55%)	55 (58%)	0.63
Clinical Features				
Baseline mRS	1 (0–2)	1 (0–2)	1 (0–1)	0.19
NIHSS on admission	9 (4–16)	8 (3–15)	15 (10–38)	< 0.001
Arterial hypertension [n (%)]	749 (91%)	662 (91%)	87 (92%)	0.75
Atrial fibrillation [n (%)]	212 (26%)	188 (26%)	24 (25%)	0.92
Coronary artery disease [n (%)]	228 (28%)	203 (28%)	25 (26%)	0.77
Oral anticoagulation [n (%)]	167 (20%)	149 (20%)	18 (19%)	0.74
Antiplatelets [n (%)]	239 (29%)	214 (29%)	25 (26%)	0.55
Antihypertensive therapy [n (%)]	521 (63%)	467 (64%)	54 (57%)	0.18
Length of stay [days]	11 (7–16)	10 (7–15)	18 (12–24)	< 0.001
Osmotic agents				
Mannitol [n (%)]	95 (12%)			
Begin/End Mannitol*	2 (1–3)/9 (6–12)	-	2 (1–3)/9 (6–12)	
Duration Mannitol [days]^†^	7 (5–10)	-	7 (5–10)	
Hypertonic saline [n (%)]	48 (6%)	14 (2%)	34 (36%)	< 0.001
Begin/End Hypertonic Saline*	3 (2–5)/9 (6–15)	2 (2–5) / 10 (7–16)	2 (1–5)/8 (6–11)	
Duration Hypertonic Saline [days]^†^	6 (4–9)	8 (4–10)	6 (4–7)	
Max. serum osmolar gap days 2–3 [mosm/kg]	8 (4–12)	7 (4–12)	14 (9–22)	< 0.001
Max. serum osmolar gap days 4–6 [mosm/kg]	9 (4–14)	8 (3–11)	15 (9–22)	< 0.001
Max. serum osmolar gap days 7–9 [mosm/kg]	10 (4–15)	9 (3–13)	15 (7–20)	< 0.001
Max. serum osmolar gap days 10–12 [mosm/kg]	9 (5–12)	8 (4–12)	10 (6–13)	0.09
Max. serum osmolality days 2–3 [mosm/kg]	292 (287–300)	292 (287–298)	306 (294–315)	< 0.001
Max. serum osmolality days 4–6 [mosm/kg]	299 (291–309)	297 (290–306)	311 (301–319)	< 0.001
Max. serum osmolality days 7–9 [mosm/kg]	299 (292–311)	298 (291–309)	307 (298–319)	< 0.001
Max. serum osmolality days 10–12 [mosm/kg]	298 (291–313)	298 (292–312)	299 (291–318)	0.99
Functional Outcome				
Discharge mRS	4 (3–5)	4 (3–5)	5 (5–5)	< 0.001
90 Day mRS (*N* = 772)	4 (2–5)	3 (2–5)	5 (4–6)	< 0.001
Poor Outcome Day 90 (mRS 4–6) [n (%)]	411 (53%)	335 (46%)	76 (80%)	< 0.001
In-hospital Mortality [n (%)]	81 (10%)	58 (8%)	23 (24%)	< 0.001

**Table 2:** Imaging characteristics of the overall cohort and those differentiated by mannitol treatment. Data are given as n (%), median (interquartile range). ICH = intracerebral hemorrhage, PHE = perihemorrhagic edema, IVH = intraventricular hemorrhage. *Refers to the day after admission (admission = day 1). ^†^Difference between peak PHE [mL] and baseline PHE [mL]. ^‡^Difference between peak PHE [mL] and baseline PHE [mL] divided by the number of days between peak PHE volume and baseline PHE volume. P-values refer to comparisons between patients with and without mannitol treatment. ^§^Five patients showed a deep location with an additional brainstem extension

	Whole Cohort (*n* = 826)	No Mannitol (*n* = 731)	Mannitol (*n* = 95)	*P* Value
Location				
Lobar [n (%)]	341 (41%)	309 (42%)	32 (34%)	0.11
Deep [n (%)]^§^	386 (47%)	336 (46%)	50 (53%)	0.22
Brainstem [n (%)]^§^	42 (5%)	38 (5%)	4 (4%)	0.68
Cerebellar [n (%)]	62 (8%)	51 (7%)	11 (12%)	0.11
Left hemisphere [n (%)]	461 (56%)	413 (56%)	48 (51%)	0.27
Imaging data				
ICH volume on admission [mL]	11.06 (4.13–25.62)	9.62 (3.58–21.14)	31.65 (16.23–54.29)	< 0.001
Peak ICH volume [mL]	13.69 (5.25–31.77)	11.69 (4.62–27.68)	42.01 (19.18–70.43)	< 0.001
Day of peak ICH volume*	1 (1–2)	1 (1–2)	2 (1–3)	< 0.001
Any secondary hematoma expansion up to 72 h [n (%)]	356 (43%)	303 (41%)	53 (56%)	0.008
Secondary hematoma expansion volume up to 72 h [mL]	1.74 (0.56–6.05)	1.63 (0.53–5.04)	5.29 (0.71–13.65)	< 0.001
IVH on admission [n (%)]	317 (38%)	258 (35%)	59 (62%)	< 0.001
IVH on follow up CT [n (%)]	373 (45%)	301 (41%)	72 (76%)	< 0.001
Peak GRAEB score	0 (0–4)	0 (0–3)	3 (1–8)	0.03
PHE volume on admission [mL]	10.565 (4.72–22.92)	9.86 (4.41–20.49)	22.92 (8.85–38.75)	< 0.001
Peak PHE volume [mL]	20.18 (7.5-40.41)	18.52 (6.87–35.7)	48.14 (22.15–81.06)	< 0.001
PHE growth up to peak volume [mL]^†^	4.63 (0.63–16.3)	3.68 (0.11–14.19)	21.88 (6.66–43.94)	< 0.001
PHE growth up to peak volume per day [mL] ^‡^	1.10 (0.11–2.94)	0.93 (0.05–2.68)	2.77 (1.22–4.62)	< 0.001
Day of peak PHE volume*	3 (1–8)	3 (1–6)	9 (2–13)	< 0.001
Number CT scans in total	3 (2–5)	3 (2–4)	6 (4–8)	< 0.001
Day of Last CT scan*	6 (2–12)	6 (2–11)	14 (7–19)	< 0.001

### PHE progression

#### PHE growth up to peak volume and peak PHE volume

**Fig. 1:**
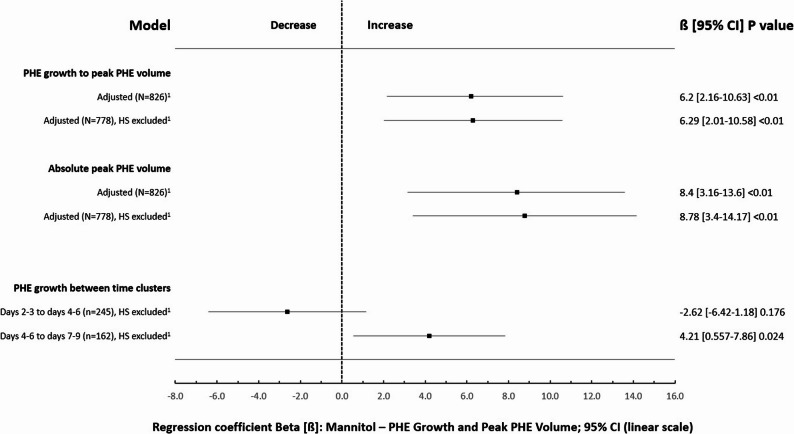
Linear association of prolonged prophylactic mannitol administration with absolute perihemorrhagic edema (PHE) growth up to peak volume (defined as the difference between peak PHE volume and admission PHE volume; primary outcome), absolute peak PHE volume (secondary outcome) and time dependent PHE growth (secondary outcome) between days 2-3 and days 4-6 as well as between days 4-6 and days 7-9 (pre-defined time-cluster; secondary outcome). ^1^Adjusted for ICH volume. HS excluded = exclusion of patients who received hypertonic saline.

In the overall cohort (N = 826), the linear regression model (adjusted for peak ICH volume) revealed an association of mannitol with increased PHE growth up to peak volume (primary outcome; b = 6.2, 95% CI 2.16–10.63, p = 0.003; ANOVA: corr. R²=0.289, F(5,820) = 68.05, p < 0.001). Excluding patients who received HS (N = 778) yielded similar results (b = 6.29, 95% CI 2.01–10.58, p = 0.004; ANOVA: corr. R²=0.264, F(4,773) = 70.52, p < 0.001). Similarly, mannitol use was associated with increased absolute peak PHE volume (secondary outcome) in the overall cohort (N = 826; b = 8.4, 95% CI 3.16–13.6, p < 0.01; ANOVA: corr. R²=0.526, F(5,820) = 184.37, p < 0.001) and in the sensitivity analysis without patients who received HS (N = 778; b = 8.78, 95% CI 3.4-14.17, p = 0.001; ANOVA: corr. R²=0.524, F(4,773) = 214.79, p < 0.001) adjusted for peak ICH volume. Please refer to Fig. 1 for a graphical overview of the aforementioned results.

The association with daily PHE growth up to peak PHE volume (secondary outcome) did not show an association with mannitol treatment adjusted for peak ICH volume (b=-0.962, 95% CI -2.12-0.195, *p* = 0.103; ANOVA: corr. R²=0.193, F(2,823) = 99.871, *p* < 0.001; without patients who received HS: b=-0.741, 95% CI -2.14-0.657, *p* = 0.299; ANOVA: corr. R²=0.203, F(2,775) = 100,158, *p* < 0.001).

#### Time-dependent associations with PHE growth

After excluding patients with HS treatment, linear regression analysis adjusted for peak ICH volume revealed a slight trend towards a negative association of mannitol administration with edema evolution (i.e. reduced PHE development) shortly after initiation of mannitol therapy (PHE development between days 2–3 and days 4–6: b=-2.62, 95% CI -6.423-1.183, *p* = 0.176; ANOVA: corr. R²=0.03, F(2,242) = 4.776, *p* = 0.009; *n* = 245). At later timepoints, mannitol administration showed an association with increased edema evolution (between days 4–6 and 7–9: b = 4.21, 95% CI 0.557–7.86, *p* = 0.024; ANOVA: corr. R²=0.04, F(2,159) = 4.384, *p* = 0.014 (*n* = 162, please see also Fig. 1). There was no association of the serum osmolar gap at any time point up to day 12 with a reduced peak PHE volume. Instead, a greater serum osmolar gap on days 4–6 and 10–12 predicted larger peak PHE volumes adjusted for peak ICH volumes (see Table 3). Serum osmolality was associated with reduced peak PHE volumes only on days 2–3 in treated patients, while no associations on any other day or in the complete cohort were found (see Table 3).

**Table 3:** Linear associations of the serum osmolar gap and serum osmolality (mosm/kg) with peak perihemorrhagic edema volume adjusted for peak intracerebral hemorrhage volume in the complete cohort and in patients treated with mannitol only. Maximum (max.) values within the indicated time cluster (days 2–3, 4–6, 7–9 and 10–12) were used. Patients treated with hypertonic saline (HS) and patients with missing data were excluded from this analysis

Complete Cohort (patients treated with HS were excluded)	*N*=	Regression- coefficient	95% CI	*P* Value
Max. serum osmolar gap days 2–3	407	-0.016	-0.06–0.03	0.462
Max. serum osmolar gap days 4–6	146	0.493	0.07–0.92	0.024
Max. serum osmolar gap days 7–9	93	0.302	-0.11–0.72	0.153
Max. serum osmolar gap days 10–12	74	0.795	0.13–1.46	0.019
Max. serum osmolality days 2–3	486	-0.021	− 0.04 − 0.003	0.087
Max. serum osmolality days 4–6	170	-0.008	− 0.19 − 0.17	0.931
Max. serum osmolality days 7–9	117	0.028	− 0.16–0.21	0.763
Max. serum osmolality days 10–12	87	-0.184	− 0.45 − 0.08	0.168
Patients with mannitol treatment only				
Max. serum osmolar gap days 2–3	48	-0.388	-1.19–0.42	0.377
Max. serum osmolar gap days 4–6	37	0.397	-0.827–1.16	0.514
Max. serum osmolar gap days 7–9	27	0.248	-0.55–1.05	0.527
Max. serum osmolar gap days 10–12	23	0.624	-0.81–2.06	0.374
Max. serum osmolality days 2–3	48	-0.502	-0.97 - -0.04	0.035
Max. serum osmolality days 4–6	38	-0.139	-0.81–0.53	0.677
Max. serum osmolality days 7–9	28	0.154	-0.27–0.58	0.459
Max. serum osmolality days 10–12	24	-0.033	-0.73–0.67	0.923

#### PS-matched cohort (PS-Match Edema)

**Fig. 2:**
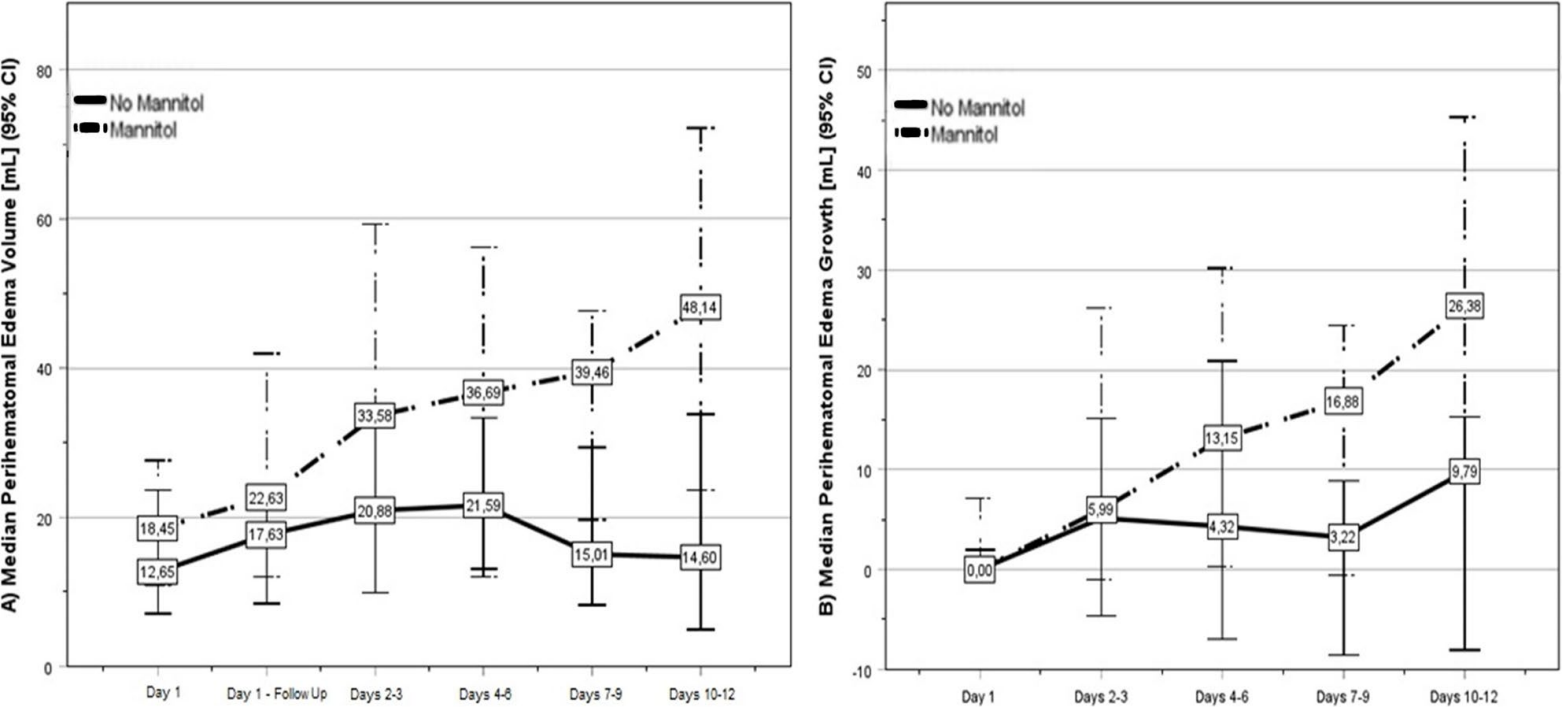
**A** Perihemorrhagic edema (PHE) evolution up to day 12 in patients with and without prolonged prophylactic mannitol administration (propensity score (PS) Match Edema accounting for peak intracerebral hemorrhage (ICH) volume and age, number of CT scans and day of last CT scan; n=116; patients without a complete dataset up to days 10-12 were excluded from the analysis for this figure). **B** Development of PHE growth up to day 12 in patients with and without prolonged prophylactic mannitol administration (PS Match Edema accounting for peak ICH volume, age, day of last CT scan and number of CT scans; n=116; patients without a complete dataset up to days 10-12 were excluded from the analysis for this figure). For this figure, PHE growth was defined as the difference between the PHE volume measured in the respective time cluster and the admission PHE volume. Patients with additional HS treatment or brainstem locations were excluded. The continuous lines indicate no-mannitol use, and the broken lines indicate mannitol use. Day 1 = admission

Table 4 shows the patient and imaging characteristics of the PS-Match Edema cohort (*n* = 116; matched for peak ICH volume and age). Although the median baseline hematoma volume was greater in the mannitol-group than in the non-mannitol group, maximum hematoma volume and secondary hematoma expansion volume, which account for peak PHE volume and PHE growth [4], did not differ between patients with and without mannitol treatment (see Table 4). The peak hematoma volume occurred later in the mannitol group. While the baseline PHE volume did not differ between patients with and without mannitol treatment, PHE growth from admission to peak volume was greater in the mannitol group. Furthermore, the peak PHE volume was greater and occurred later in the mannitol group (see Table 4). Figure 2 depicts PHE development in patients with and without mannitol treatment in the PS-matched cohort up to days 10–12: Especially at later time-points, PHE growth and volumes seem to be increased in patients with mannitol treatment.

**Table 4:** PS Match Edema: Clinical, patient and imaging characteristics in the PS-matched cohort accounting for peak ICH volume, age, number of CT scans and day of last CT scan. Patients with additional HS treatment or brainstem locations were excluded. Data are given as n (%), median (interquartile range). PHE indicates perihemorrhagic edema. IVH: intraventricular hemorrhage. PS propensity score. *Refers to the day after admission (admission = day 1). ^†^Refers to days of treatment. ^‡^Difference between peak PHE [mL] and baseline PHE [mL]. ^§^Difference between peak PHE [mL] and baseline PHE [mL] divided by the number of days between peak PHE volume and baseline PHE volume

	No Mannitol (*n* = 58)	Mannitol (*n* = 58)	*P* Value
Demographics			
Age [years]	74 (66–79)	72 (59–77)	0.13
Male [n (%)]	34 (59%)	35 (60%)	0.85
Clinical Features			
Baseline mRS	1 (0–2)	1 (0–1)	0.90
NIHSS on admission	9 (6–17)	15 (10–24)	0.03
Arterial hypertension [n (%)]	52 (90%)	56 (97%)	0.14
Atrial fibrillation [n (%)]	18 (31%)	14 (24%)	0.41
Coronary artery disease [n (%)]	19 (33%)	13 (22%)	0.22
Oral anticoagulation [n (%)]	19 (33%)	12 (21%)	0.14
Antiplatelets [n (%)]	17 (29%)	12 (21%)	0.28
Antihypertensive therapy [n (%)]	41 (71%)	31 (53%)	0.06
Length of Stay [days]	14 (7–22)	17 (12–23)	0.05
Osmotic agents			
Begin/End Mannitol*		2 (1–4)/9 (7–11)	
Duration Mannitol^†^		7 (5–9)	
Functional Outcome			
Discharge mRS	5 (4–5)	5 (5–5)	0.07
90 Day mRS	4 (3–6)	5 (4–6)	0.33
In-hospital Mortality [n (%)]	11 (19%)	11 (19%)	0.99
Location			
Lobar [n (%)]	30 (51.7%)	16 (27.6%)	0.008
Deep [n (%)]	27 (47%)	36 (62%)	0.09
Cerebellar [n (%)]	1 (7%)	6 (10%)	0.05
Left hemisphere [n (%)]	31 (53%)	27 (47%)	0.46
Imaging Data			
ICH volume on admission [mL]	17.42 (7.87–35.09)	31.17 (13.76–53.28)	0.014
Peak ICH volume [mL]	25.95 (11.46–54.91)	39.44 (16.27–62.34)	0.11
Day of peak ICH volume*	1 (1–2)	2 (1–3)	0.05
Any secondary hematoma expansion up to 72 h [n (%)]	*N* = 29 (50%)	*N* = 30 (51%)	0.85
Any secondary hematoma expansion up to 72 h [mL]	4.31 (1.75–10.8)	4.11 (0.71–10.98)	0.33
IVH on admission [n (%)]	26 (45%)	40 (69%)	0.009
IVH on follow up CT [n (%)]	34 (59%)	51 (88%)	< 0.001
Peak GRAEB score	2 (0–7)	4 (2–7)	0.03
PHE volume on admission [mL]	12.7 (6.88–31.37)	21.18 (9.43–41.99)	0.08
Peak PHE volume [mL]	35.24 (19.73–52.7)	45.69 (23.95–78.19)	0.04
PHE growth up to peak volume [mL] ^‡^	11.9 (1.37–25.84)	21.49 (9.17-42.76)	0.03
PHE growth up to peak volume per day [mL]^§^	1.47 (0.64–4.74)	2.56 (1.22–4.21)	0.304
Day of peak PHE volume*	3 (1–12)	9 (4–14)	0.03
Number CT scans in total	4 (2–7)	6 (4–8)	0.04
Day last CT scan*	11 (3–17)	14 (8–17)	0.19

### Functional outcome

**Fig. 3:**
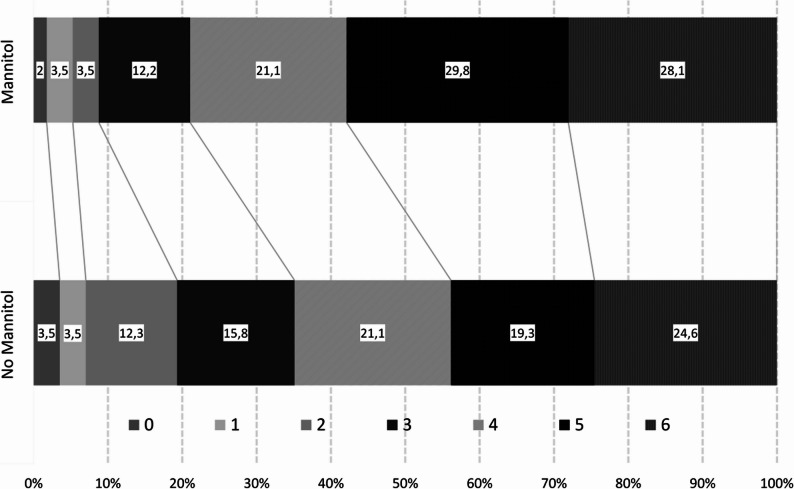
Distribution of day 90 outcome according to the modified Rankin Scale (mRS; after propensity score (PS) Match Outcome, accounting for peak intracerebral hemorrhage (ICH) volume, age, baseline mRS, baseline National Institutes of Health Stroke Scale (NIHSS), number of CT scans and day of last CT scan); n=114; mannitol group: n=57; non-mannitol group: n=57; patients with additional hypertonic saline (HS) treatment or brainstem location were excluded; see also Table 4

**Fig. 4:**
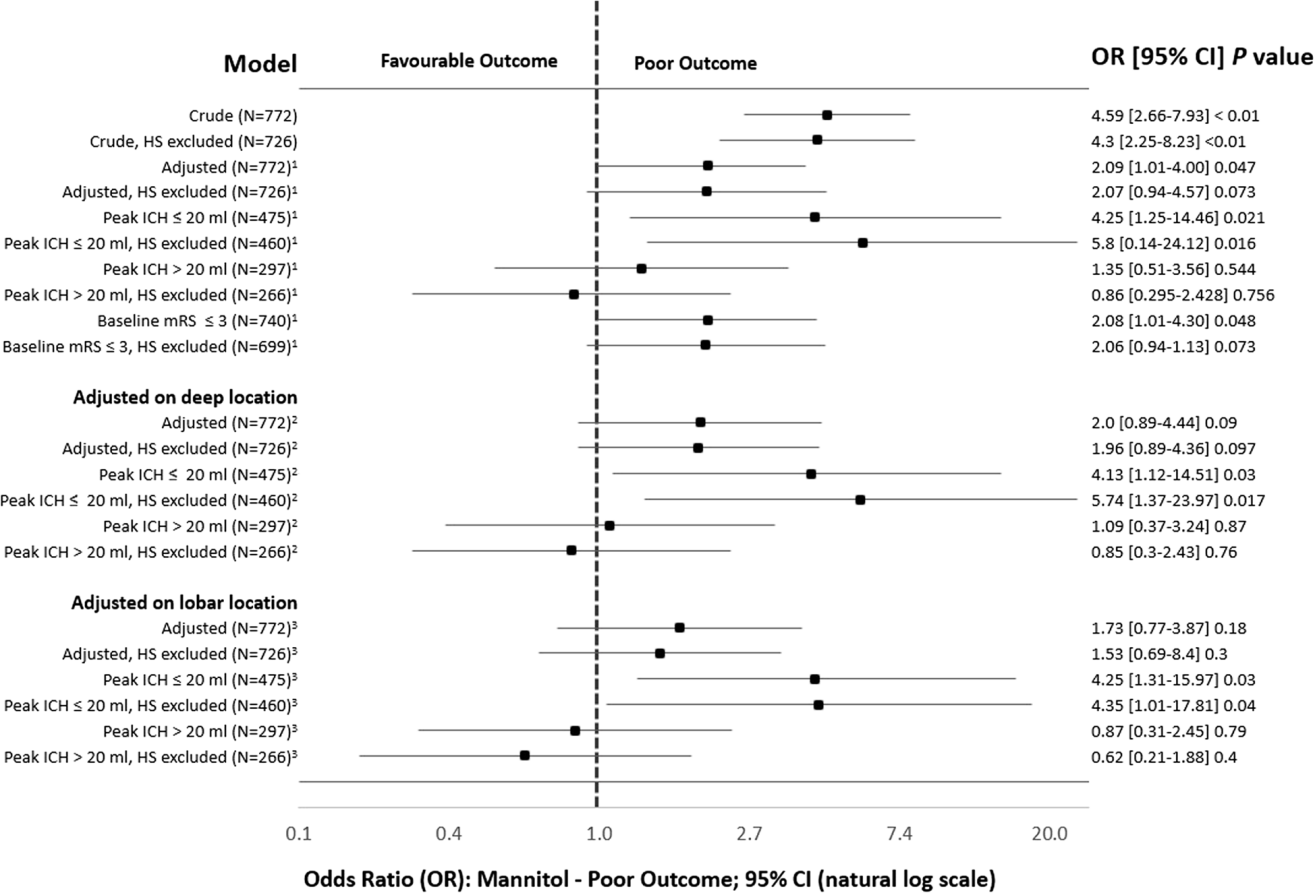
Association of prolonged prophylactic mannitol administration with poor outcome (modified Rankin Scale (mRS) score of 4-6) on day 90. Data from the overall cohort (n=772 with available outcome data), dichotomized according to hematoma volume ( 20mL) and in patients with a baseline mRS ≤3 only (sensitivity analyses). The solid boxes represent estimates of the treatment effect on the risk of outcomes. The horizontal lines represent the 95% confidence intervals (CIs). HS excluded = exclusion of patients who received hypertonic saline. OR = odds ratio. ^1^Adjusted for age, baseline mRS score, peak intracerebral hemorrhage (ICH) volume, peak perihemorrhagic edema (PHE) volume, peak GRAEB score, and the use of hypertonic saline score and the use of hypertonic saline (unless HS excluded). ^2^Adjusted for age, baseline mRS score, peak ICH volume, peak PHE volume, peak GRAEB score, use of hypertonic saline, and deep location and the use of hypertonic saline (unless HS excluded). ^3^Adjusted for age, baseline mRS score, peak ICH volume, peak PHE volume, peak GRAEB score, use of hypertonic saline, and lobar location and the use of hypertonic saline (unless HS excluded).

Owing to the mentioned substantial imbalances between treatment groups, we focused on results from the PS-matched cohort (PS Match Outcome, *n* = 114) and adjusted logistic regression analyses (*n* = 772 with available outcome data). After PS-matching the following variables relevant for outcome prediction in ICH patients were evenly distributed between the two treatment groups: age, baseline mRS score, baseline NIHSS score, secondary hematoma expansion up to 72 h, IVH at follow-up, peak GRAEB score, and location distribution. The peak ICH volume still showed a trend towards larger volumes in patients treated with mannitol (see Tables 5 and 21 (IQR 10–60) mL vs. 39 (IQR 16–59) mL, *p* = 0.052). The long-term outcome did not differ significantly in the PS matched cohort, whereas the estimates revealed poorer outcomes in patients treated with mannitol (median mRS score day 90, 4 (IQR 3–5) and 5 (IQR 4–6), *p* = 0.14; see Table 5, “PS Match Outcome”). Furthermore, there was a trend toward a higher rate of poor outcomes in patients treated with mannitol (79% vs. 65% respectively, *p* = 0.097, see Fig. 3 for the distribution of outcomes in the PS-matched cohort). In the overall cohort, the logistic regression analysis (see Fig. 4) suggested an adverse effect of mannitol on long-term outcome (crude (*n* = 772) OR = 4.59, 95% CI 2.66–7.93, *p* < 0.001) and after adjustment (*n* = 772, adjusted OR = 2.09, 95% CI 1.01-4.00, *p* = 0.047). This effect remained stable even after exclusion of patients with a baseline mRS score > 3 (*n* = 744). Additional analyses adjusted for deep or lobar location yielded similar results (see Fig. 4). Notably, mannitol was associated with poor outcome in patients with smaller ICHs (< 20 mL, *n* = 475; OR = 4.25, 95% CI 1.25–14.46, *p* = 0.021) whereas no such association was detected in patients with ICH volumes > 20 mL (*n* = 297; OR = 1.35, 95% CI 0.51–3.45, *p* = 0.544). Excluding patients, who received HS yielded similar results, see Fig. 4.

**Table 5:** Clinical, patient and imaging characteristics in the PS-matched cohort (PS Matching Outcome). PS matching accounted for peak ICH volume, age, the baseline mRS score and the baseline NIHSS score, the number of available CT scans during the in-hospital stay and the day of the last available CT scan. Patients with additional HS treatment or brainstem locations were excluded. GCS: Glasgow Coma Scale [25], ICH: intracerebral hemorrhage, IVH: intraventricular hemorrhage, NIHSS: National Institute of Health Stroke Scale, mRS: modified Rankin Scale, PHE: perihemorrhagic edema. *Refers to the day after admission (admission = day 1). ^†^Refers to days of treatment. ^‡^Difference between peak PHE [mL] and baseline PHE [mL]

	No Mannitol (*n* = 57)	Mannitol (*n* = 57)	*P* Value
Demographics			
Age [years]	73 (64–78)	72 (59–77)	0.56
Male [n (%)]	34 (60%)	34 (60%)	0.99
Clinical Features			
Baseline mRS	0 (0–1)	1 (0–1)	0.71
NIHSS on admission	14 (5–21)	15 (10–24)	0.3
Arterial Hypertension [n (%)]	53 (93%)	55 (97%)	0.38
Atrial fibrillation [n (%)]	21 (37%)	14 (25%)	0.16
Coronary artery disease [n (%)]	14 (25%)	13 (23%)	0.83
Oral anticoagulation [n (%)]	18 (32%)	12 (21%)	0.2
Antiplatelets [n (%)]	19 (33%)	11 (19%)	0.09
Antihypertensive therapy [n (%)]	36 (64%)	30 (53%)	0.26
Length of stay [days]	14 (7–21)	18 (12–21)	0.03
Imaging-Data			
ICH volume on admission [mL]	14.93 (7.15–35.09)	30.69 (13.76–50.64)	0.001
Peak ICH volume [mL]	21.01 (10.02–59.95)	38.95 (16.27–59.31)	0.05
Day of peak ICH volume*	1 (1–2)	2 (1–3)	0.05
Any secondary hematoma expansion up to 72 h [n (%)]	*N* = 29 (51%)	*N* = 30 (53%)	0.85
Any secondary hematoma expansion up to 72 h [mL]	4.36 (0.91–13.79)	4.11 (0.71–10.98)	0.51
IVH on admission [n (%)]	26 (46%)	40 (70%)	0.009
IVH on follow up CT [n (%)]	35 (64%)	50 (88%)	0.4
Peak GRAEB score	3 (0–7)	4 (2–7)	0.12
PHE volume on admission [mL]	14.99 (7.87–27.49)	21.05 (9.43–40.49)	0.13
Peak PHE volume [mL]	32.8 (12.04–62.22)	45.18 (23.95–74.76)	0.05
PHE growth up to peak volume [mL] ^‡^	8.06 (10.02–59.95)	38.95 (16.27–59.31)	0.01
Day of peak PHE volume*	2 (2–11)	9 (4–14)	0.001
Number CT scans in total	5 (2–7)	6 (4–8)	0.03
Day Last CT scan*	10 (3–17)	10 (3–17)	0.07
Location			
Lobar [n (%)]	22 (39%)	15 (26%)	0.16
Deep [n (%)]	33 (58%)	36 (63%)	0.57
Cerebellar [n (%)]	2 (4%)	6 (11%)	0.14
Left hemisphere [n (%)]	32 (56%)	26 (46%)	0.26
Osmotic agents			
Begin Mannitol/End Mannitol*	-	2 (1–4)/9 (7–11)	
Duration Mannitol [days]^†^	-	7 (5–9)	
Functional Outcome			
Discharge mRS	5 (4–5)	5 (5–5)	0.06
90 Day mRS	4 (3–5)	5 (4–6)	0.14
Poor Outcome Day 90 (mRS 4–6) [n (%)]	36 (65%)	45 (79%)	0.097
In-hospital mortality [n (%)]	7 (12%)	11 (19%)	0.3

## Discussion

Prophylactic administration of mannitol over several days did not achieve the intended effect of reducing PHE volume or its progression. We could not find a beneficial effect of prophylactic mannitol use over several days on long-term outcome or PHE development in our large ICH cohort, especially in patients with smaller ICH volumes (< 20mL). In all analyses, estimates pointed towards worse functional outcomes in patients treated with mannitol. While for smaller ICH volumes < 20mL the unfavorable effect seemed more pronounced in our dataset, a clear positive effect could also not be found for larger ICH volumes > 20mL. However, based on our data, we cannot exclude that single doses or a short-term administration of mannitol up to 72 h after onset may have a beneficial effect on PHE development. The retrospective nature of the analysis and considerable imbalances between treatment groups preclude definitive conclusions.

Interestingly, we observed the most pronounced increase in PHE volume in patients treated with mannitol at later time points, particularly from days 4 to 6 onward, when mannitol had already been administered for several days. In the non-mannitol group, the PHE volume showed an earlier peak PHE volume at days 4–6 and a consecutive volume decrease afterwards, as depicted in Fig. 4. These observations as well as the results depicted in Tables 2 and 3 suggest a prolonged and more intense increase in edema in the mannitol group. While this observation is merely descriptive without proven causality as discussed in the limitation section below, pathophysiological data support these findings. A possible explanation for the increased PHE volume in patients treated with prophylactic mannitol might be an accumulation of mannitol in the brain tissue due to disruption of the blood-brain barrier and a consecutively established reverse gradient that draws water back into the extracellular space rather than removing it [9, 11, 12]. As further support of this hypothesis, we found an association of the serum osmolar gap with increased peak PHE volumes at later time points, whereas estimates pointed towards a decreased peak PHE volume only when serum osmolality of treated patients on days 2–3 (= early administration phase) was included. In addition, we found a slight trend towards an association of mannitol administration with reduced PHE development adjusted for ICH volume shortly after initiation of mannitol treatment (up to days 4–6). Again, at later time-points, this effect reversed. Thus, after all we cannot exclude that a short term or single bolus administration of mannitol may have a beneficial effect on PHE evolution. This effect of a short-term mannitol administration (single dose and up to 24 h) is currently studied in a UK-based phase II randomized, controlled trial [26].

Since peak PHE volume and early PHE expansion [2, 3, 6, 7] are considered independent predictors of functional outcome [8, 10], this might be a possible pathophysiological link to our findings regarding functional outcome in patients treated with prophylactic mannitol use. In particular, the adjusted logistic regression models in the overall cohort revealed an unfavorable effect of mannitol. However, a clear dose-response relationship in general could not be shown in our setting. Furthermore, from a pathophysiological point of view, it remains to be elucidated whether the abovementioned water movement mediates the association with outcome rather than a direct toxic effect or an additional inflammatory response initiated by mannitol after it crosses the blood-brain-barrier. At least, existing results suggest that toxic and inflammatory processes associated with PHE development on brain imaging may explain the association with functional outcome [4, 27] rather than mere movement of water or fluids. However, while our data do not support the prophylactic (long-term) use of mannitol in ICH patients, we did not assess the association of mannitol boluses with acute intracranial pressure elevation [28].

Existing studies support our findings regarding clinical outcome in general. A retrospective analysis by Shah et al. analyzing data from 608 patients from the ERICH dataset revealed no evidence of improved outcome or survival rates in patients receiving hyperosmolar therapy (mannitol and/or HS) [29]. There was no evaluation of PHE in this study. A prospective cohort including 805 stroke and ICH patients revealed no effect of routine prophylactic mannitol treatment on the case fatality rate after 30 days and one year [30]. Again, no PHE assessment was performed in this study. Furthermore, the outcomes did not include functional outcome and both ischemic stroke patients and ICH patients were included. Similarly, Wang et al. reported no improvement in functional outcome in patients treated with mannitol in the INTERACT-2-dataset (*n* = 2.526 with 1533 patients receiving mannitol) [31]. The authors also did not assess PHE development. While both Shah et al. and Wang et al. included the mRS score on day 90 as an outcome measure, these studies did not account for individual treatment regimens regarding the beginning, duration and dosage of mannitol or serum osmolality in their analysis [29, 31]. However, Misra et al. described a specific dosing regimen in a small study: mannitol was administered over 7 days with a tapering scheme after 5 days but without dose adjustment according to serum osmolality. The authors also reported no effect of prophylactic mannitol treatment on 1-month mortality or the 3-month Barthel Index in their prospective randomized trial (*n* = 128, 65 patients who received mannitol) [32]. Again, no PHE assessment was performed. To conclude, in general some evidence suggests no benefit of prophylactic mannitol use, but several methodological issues, as mentioned above, limit the generalizability of the results. Furthermore, the association of mannitol treatment with edema evolution has not been assessed thus far.

We observed, that especially in patients with a hematoma volume < 20mL, a long-term prophylactic mannitol administration is associated with a poor outcome. However, also in larger ICH volumes, the estimates pointed towards a poor outcome, while the 95% CI also included a possible positive outcome effect. It is well established that large hematoma volumes are associated with a poor outcome in general [21]. Thus, a possible additional harmful effect of a long-term mannitol administration may not be as evident in these patients as in patients with smaller ICH volumes, which are associated with better outcomes in general.

Our study has several strengths: First, in addition to long-term functional outcome we analyzed long-term PHE data that cover not only the acute phase, but also the entire in-hospital stay (up to 35 days). This is important, since PHE growth might continue up to 14 days after ictus [33] or even longer [34]. Furthermore, this allows an assessment of PHE development following repeated mannitol administration over several days. Second, we could include a large dataset, which allowed adjustments for several relevant confounding factors. Third, to achieve observer-independent results, a validated semiautomatic volumetric assessment was used to obtain PHE and ICH volumes, which proved reliable results compared with those of MRI-imaging and reduced observer related errors [16]. Finally, the most important factor associated with PHE evolution is hematoma volume [4]. To assess factors associated with PHE development besides hematoma volume, a rigorous adjustment for hematoma volume, as performed in our study, is necessary. Furthermore, several attempts have been made to minimize this association in order to establish an edema measure independent from hematoma volume. The most promising approaches seem to be the relative PHE / PHE ratio (rPHE; corresponds to the absolute PHE volume divided by the hematoma volume) and the edema extension distance (EED; corresponds to the approximate radius of the PHE expansion, assuming a spherical shape) [35, 36]. However, relative PHE shows an inverse association with hematoma volume in general [33, 37], while the EED was not independent from hematoma volume in our cohort (data not shown). Furthermore, recent data demonstrated that absolute PHE volume shows a stronger association with functional outcome than EED [38]. Thus, we decided to use peak PHE volume and evolution up to peak PHE instead of relative PHE and EED, and rigorously adjusted all analyses for ICH volume.

Limitations of our study include the retrospective and monocentric study design: in particular, confounding by indication regarding early prophylactic mannitol treatment should be considered, since the decision to initiate treatment has likely been associated with an earlier PHE increase or a larger ICH volume on admission in the mannitol group. Therefore, a causal interpretation of the observed association between excessive PHE increase and mannitol treatment is not possible in our cohort. Moreover, the treatment protocols allowed for variability in mannitol dosing and treatment duration, including the adjunctive administration of hypertonic saline. Furthermore, several relevant imbalances were detected in the treatment groups. However, we performed adjustments, including multivariable regression analysis and PS matching, to account for those imbalances. Yet, some residual baseline imbalances persisted. In the “PS Match Edema” cohort, baseline hematoma volume was higher in the treatment group, although peak ICH volumes and secondary hematoma expansion volumes were comparable—key determinants for valid PHE assessment [4]. As described above, EED was also associated with hematoma volume in our cohort; therefore, in line with the considerations outlined above, the use of EED instead of absolute PHE volume in our analyses would not have resolved this issue. In the “PS Match Outcome” cohort, treatment group patients presented with larger admission and peak hematoma volumes; however, secondary hematoma expansion rates/volumes and baseline NIHSS and mRS scores were well balanced. Furthermore, in the overall cohort, multivariable logistic regression models, adjusted for all residual imbalances, consistently confirmed the associations with long-term functional outcomes. To assess possible dose-dependent effects, we used serum osmolality and the osmolar gap instead of mannitol doses, which may limit the reliability of this assessment. Since the dosing was adapted on the basis of serum osmolality and most patients received equal dosing regimens, we considered this approach more reliable in our cohort than assessing the dosing regimens themselves. Prospective data are needed to fully assess this issue. Several patients received hypertonic saline (HS) as an additional osmotic therapy, which may have introduced bias into the results. Therefore, all analyses involving patients treated with HS were adjusted for HS administration, and additional sensitivity analyses were performed excluding these patients. As these adjustments and sensitivity analyses did not alter the findings, we do not consider HS treatment to be a relevant confounder in our cohort. However, due to multiple testing, an increased risk of a type 1 error should be acknowledged, when interpreting our results. The time points of cerebral imaging were not standardized and differed between individual patients, which possibly impairs the comparability of PHE development between treatment groups. To account for this, time points of cerebral imaging have been merged into prespecified time clusters. Furthermore, we accounted for the frequency and time points of neuroimaging in our PS-matching algorithm and included a more standardized PHE growth measure by dividing the volume change by the time between the respective images. However, since PHE growth does not follow a linear trajectory over time [4], this measure may overestimate PHE growth in the acute phase up to 72 h, and consequently the linear regression results based on this measure must to be interpreted with caution. Thus, we cannot exclude the possibility that differences in the timing of imaging between groups may bias PHE related results. Furthermore, despite the relatively long time period of PHE monitoring mentioned above and, compared with other studies [8], the clear definition of peak PHE volume, the real peak PHE volume may still have been missed [33, 34]. Due to the retrospective nature of our study, we could not assess adverse events related to a long-term mannitol administration. However, since our data did not show any efficacy of a long-term prophylactic mannitol administration, the missing safety data does not change or weaken our conclusion. Finally, we did not assess intracranial pressure, which may also be an interesting aspect but was not the aim of the present study.

## Conclusions

In summary, prolonged prophylactic mannitol administration over several days does not seem to improve long-term outcome. In contrast, our data even suggest increased PHE development and an association with worse outcomes in patients treated with prophylactic mannitol for several days, such that even a potential harmful effect cannot be excluded. Furthermore, our data suggest, that future studies should investigate whether short-term administration during the early acute phase, up to 72 h after onset of ICH, may be associated with reduced PHE development and improved outcomes. However, owing to the abovementioned limitations, conclusions should be drawn carefully on the basis of our data. 

## Data Availability

The data that support the findings of this study are available from the corresponding author upon reasonable request.
