# Correction: Association of prophylactic mannitol administration with perihemorrhagic edema and long-term outcome in patients with intracerebral hemorrhage

**DOI:** 10.1186/s12883-026-05160-5

**Published:** 2026-07-15

**Authors:** Maximilian Kaertner, Kosmas Macha, Jochen Sembill, Anne Mrochen, Michael Knott, Stefan Lang, Hannes Lücking, Joji B. Kuramatsu, Bastian Volbers

**Affiliations:** 1 Department of Neurology, Friedrich-Alexander-Universität Erlangen-Nürnberg (FAU), Erlangen, Germany; 2 Department of Neuroradiology, Friedrich-Alexander-UniversitätErlangen-Nürnberg (FAU), Erlangen, Germany; 3 Department of Neurology, Inselspital, Bern University Hospital, University of Bern, Bern, Switzerland; 4 Department of Neurology, RoMed Klinikum Rosenheim, Rosenheim, Germany

**Correction: BMC Neurol 26, 230 (2026)**

**https://doi.org/10.1186/s12883-026-04858-w**

Following publication of the original article [1], the authors reported a typographical errors in Fig. 4 that could lead to misinterpretation. The errors do not affect the study’s results or conclusions.

Incorrect Fig. 4:


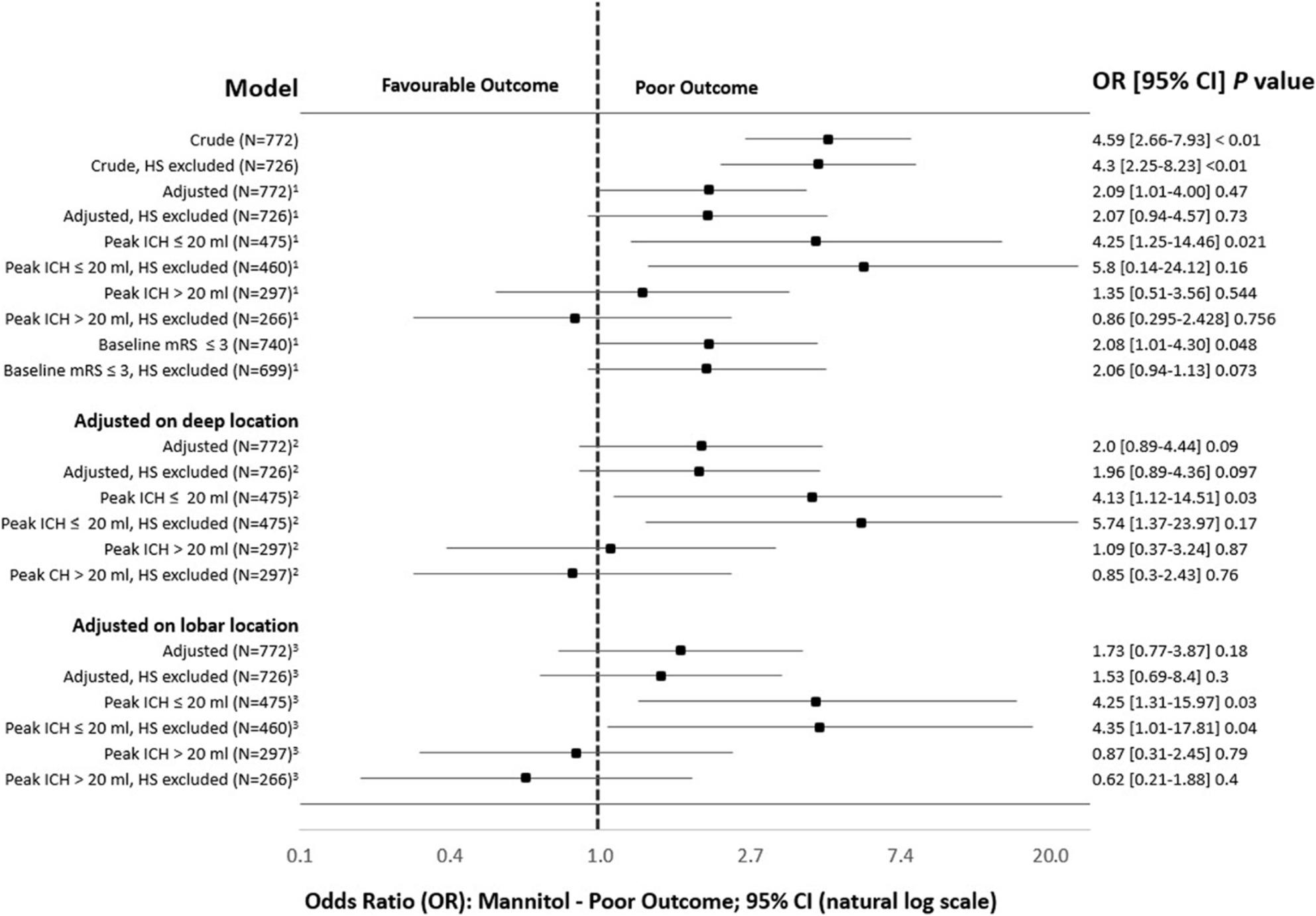


Correct Fig. 4.


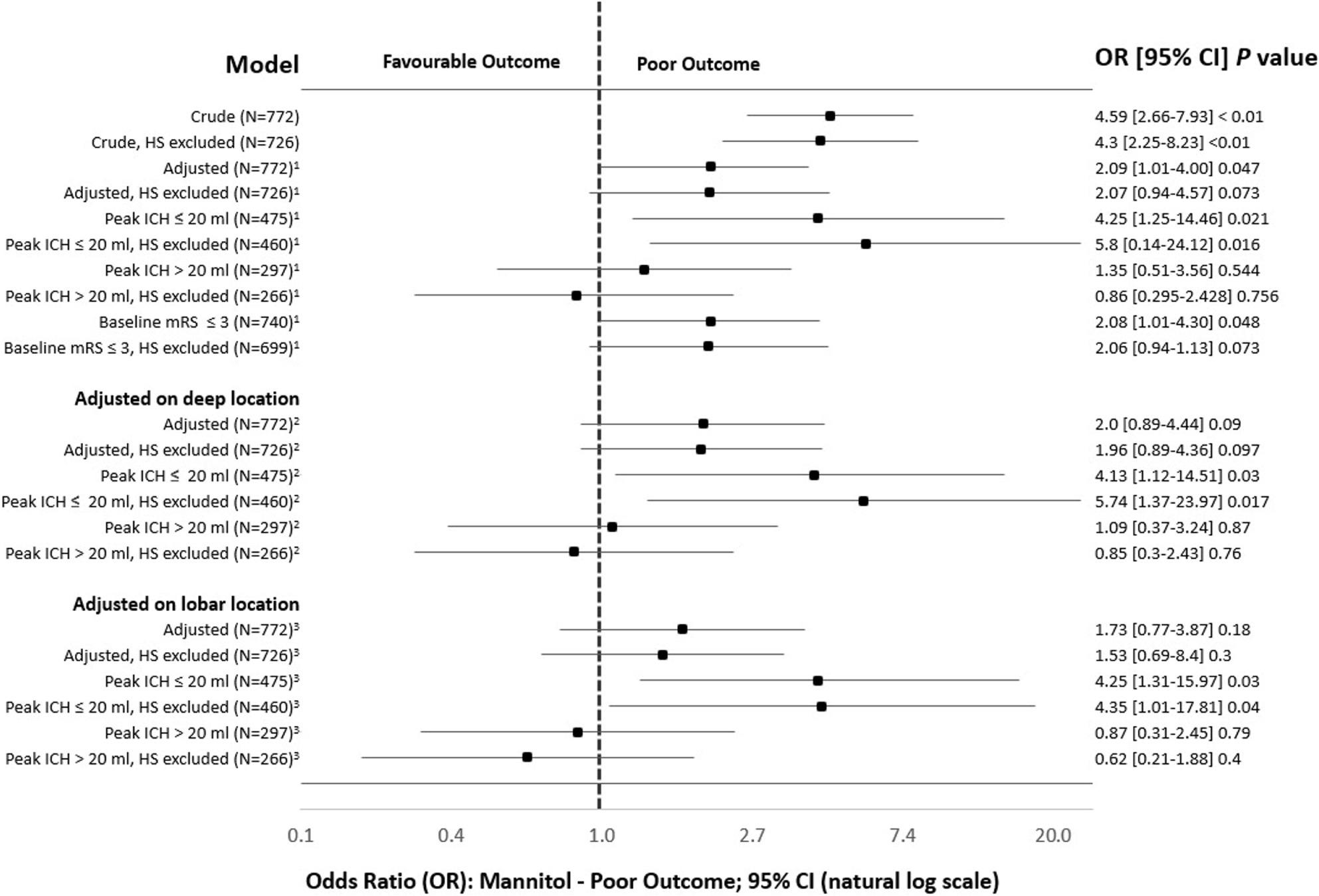


The original article [1] has been updated.
